# The efficacy of flexor tenotomy to prevent recurrent diabetic foot ulcers (DIAFLEX trial): Study protocol for a randomized controlled trial

**DOI:** 10.1016/j.conctc.2023.101107

**Published:** 2023-03-11

**Authors:** M.A. Mens, T.E. Busch-Westbroek, S.A. Bus, J.J. van Netten, R.H.H. Wellenberg, G.J. Streekstra, M. Maas, M. Nieuwdorp, G.M.M.J. Kerkhoffs, S.A.S. Stufkens

**Affiliations:** a Amsterdam UMC, Location University of Amsterdam, Radiology and Nuclear Medicine, Meibergdreef 9, Amsterdam, the Netherlands; b Amsterdam UMC, Location University of Amsterdam, Rehabilitation Medicine, Meibergdreef 9, Amsterdam, the Netherlands; c Amsterdam UMC, Location University of Amsterdam, Biomedical Engineering and Physics, Meibergdreef 9, Amsterdam, the Netherlands; d Amsterdam UMC, Location University of Amsterdam, Internal Medicine, Meibergdreef 9, Amsterdam, the Netherlands; e Amsterdam UMC, Location University of Amsterdam, Orthopaedic Surgery, Meibergdreef 9, Amsterdam, the Netherlands; f Amsterdam Movement Sciences, Rehabilitation and Development, Amsterdam, the Netherlands; g Amsterdam Cardiovascular Sciences, Diabetes and Metabolism, Amsterdam, the Netherlands

**Keywords:** Flexor tenotomy, Foot ulcer, Toe deformity, Prevention, CBCT, Cone-Beam Computed Tomography, DIPJ, Distal Interphalangeal Joint, DM, Diabetes Mellitus, IPJ, Interphalangeal Joint, MTPJ, Metatarsal Phalangeal Joint, PIPJ, Proximal Interphalangeal Joint, RCT, Randomized Controlled Trial, ROI, Region Of Interest, SD, Standard Deviation, SF-36, Short-Form-36, WTBCT, Weight-Bearing CT, μSv, Microsievert

## Abstract

Foot ulcers are a frequent and costly problem in people with diabetes mellitus and can lead to amputations. Prevention of these ulcers is therefore of paramount importance. Claw/hammer toe deformities are commonly seen in people with diabetes. These deformities increase the risk of ulcer development specifically at the (tip of) the toe. Percutaneous needle tenotomy of the tendon of the m. flexor digitorum longus (tendon tenotomy) can be used to reduce the severity of claw/hammer toe deformity with the goal to prevent ulcer recurrence. The main objective of this randomized controlled trial is to assess the efficacy of flexor tenotomy to prevent recurrence of toe ulcers in people with diabetes and a history of toe (pre-)ulcers. Additionally, we aim to assess interphalangeal joints (IPJ) and metatarsophalangeal joint (MTPJ) angles in a weight-bearing and non-weight-bearing position, barefoot plantar pressure during walking, cost-effectiveness and quality of life before and after the intervention and compare intervention and control study groups. Sixty-six subjects with diabetes and claw/hammer toe deformity and a recent history of (pre-)ulceration on the tip of the toe will be included and randomized between flexor tenotomy of claw/hammer toes (intervention) versus standard of care including orthosis and shoe offloading (controls) in a mono-center randomized controlled trial.

**Clinicaltrials.gov registration:**

NCT05228340.

## 1 Introduction

Diabetic foot ulcers are a common problem, with a global prevalence of 6.3% in people with diabetes, and are one of the biggest risk factors for lower extremity amputation [[1], [2], [3]]. The formation of these ulcers is multifactorial and causes include peripheral neuropathy, vascular deficiency and mechanical stress [4]. In peripheral neuropathy the protective sensation in the extremities, mainly in the feet, deteriorates [5]. Detection of foot trauma is diminished causing people with diabetes to neglect taking measures when risk for foot ulcers increases. Hyperglycemia impairs leukocyte and complement function, thereby increasing chances for invasive infections [6]. Micro- and macrovascular disease is a co-morbidity often seen in people with diabetes mellitus debilitating ulcer healing [7]. Diabetic foot ulcers require off-loading treatment (e.g. total contact casts) often accompanied by (long-term) antibiotics [8]. These treatments can burden people in their daily activities. When healed, these ulcers have a high recurrence rate of 40% per person per year in Europe [9,10]. Prevention of diabetic ulcers is therefore of great importance.

In people with diabetic peripheral polyneuropathy, deformities of the feet are more prevalent than in people without diabetes [11]. Common deformities are claw toe and hammer toe deformity. The exact mechanism behind these deformities in this patient group is not fully understood, but seems related to a mismatch in extensor and flexor function due to intrinsic muscle atrophy [12]. In claw/hammer toe deformity there is additional pressure either underneath the metatarsal heads or on the tip of the toe [13]. This excess pressure increases the chance of ulcer development on these places on the foot. Claw/hammer toe deformity can be treated conventionally with off-loading techniques such as orthopedic shoes or a toe orthosis [11]. This is not always sufficient since pressure points can still occur when the shoes do not fit properly or when the patient is not adherent to wearing them [14].

A surgical option for treating claw/hammer toe deformity is flexor tenotomy [15]. In this procedure, which has been practiced for many years worldwide, the long flexor tendon of the affected toe is severed. This is a minimally invasive procedure that can take place in the out-patient clinic [16]. A surgeon uses a needle to sever the tendon (duration 1–2 min) and due to the sensory loss caused by peripheral polyneuropathy anesthesia is often not necessary [17]. The procedure causes the toes to straighten, reducing the angles in the distal and proximal interphalangeal joint (DIPJ, PIPJ) and the metatarsal phalangeal joint (MTPJ), and reducing the plantar pressure [18].

The beneficial effects of flexor tenotomy in people with diabetes and claw/hammer toe deformity have been investigated in retrospective and prospective case series [[16], [17], [18], [19], [20], [21], [22]]. Currently, one randomized controlled trial is being conducted and one was published in September 2022 [23,24]. However, there is still need for more evidence to further substantiate the benefits of flexor tenotomy as well as the need for evaluation in a controlled study design of changes in the biomechanical and musculoskeletal structure of the foot due to the flexor tenotomy.

This randomized controlled trial aims to assess the efficacy of flexor tenotomy to prevent recurrent diabetic foot ulcers, the biomechanical and musculoskeletal changes due to the procedure, the changes in quality of life and the cost-effectiveness of the procedure.

## 2 Materials and methods

### 2.1 Objectives

The primary objective is to assess the efficacy of flexor tenotomy (intervention) versus standard of care (including orthosis and shoe offloading, control) on the incidence of ulcer recurrence on the toes indicated for flexor tenotomy, on the adjacent toes and on the metatarsal heads. Secondary objectives are musculoskeletal changes expressed in MTPJ, PIPJ and DIPJ angles, biomechanical changes expressed in barefoot plantar pressure during walking, quality of life, the cost-effectiveness of flexor tenotomy and adverse events of the surgery.

### 2.2 Trial design

This study concerns a randomized controlled trial (RCT) with a 24-month follow-up period at the out-patient clinic (Table 1). Sixty-six participants will be included. Inclusion started in March 2022. After informed consent is signed, participants are randomized into two groups: usual care (control) or usual care plus flexor tenotomy (intervention). The researchers analyzing the effect of the data will be blinded for group allocation. The treating physician, orthopedic surgeon and participant will not be blinded to group allocation. The flexor tenotomy will be an addition to usual care and is scheduled after randomization. This means that the intervention group will receive the same standard care as the control group including orthopedic shoes.

**Table 1:** Standard protocol items.

**Time point**	Study period						
	Enrolment	Allocation	Post-allocation				Close-out
	−2 weeks	−1 week	0	1 week	6 months	12 months	24 months
Enrolment							
Initial eligibility screen	X						
Study information to participant	X						
Initial willingness to participate		X					
Crosscheck inclusion/exclusion criteria		X					
Informed consent			X				
Final eligibility screen			X				
Allocation			X				
**Interventions**							
Usual care (both groups)			X	X	X	X	X
Flexor tenotomy (intervention group only)				X			
**Assessments**							
Demographic and disease-related characteristics			X				
Barefoot pressure			X		X	X	
Weight-bearing CT			X		X	X	
SF-36			X		X	X	X
EQ-5D-5L			X		X	X	X
Ulcer formation			X	X	X	X	X
Notes of received foot care					X	X	X
Process evaluation					X	X	X

### 2.3 Eligibility criteria

In order to be eligible to participate in this study, a participant must meet all of the following criteria.

• A minimum age of 18 years
• Sufficient understanding of Dutch/English language
• Capable of providing informed consent
• Loss of protective sensation as a result of peripheral polyneuropathy
• Diabetes mellitus type 1 or 2
• A minimum of 1 claw/hammer toe on either foot
• A documented history of diabetic (pre-)ulcers underneath the tip of the toe in the past 5 years. Pre-ulcers include abnormalities of or damage to the nail, callus formation and hematomas.

A potential participant who meets any of the following criteria will be excluded from participation in this study.

• Open ulcer(s) on the toes
• Previous participation in the study
• Pregnant women
• Concomitant participation in a study in which the participant is exposed to X-rays (due to the use of weight-bearing CT in this study)
• Critical ischemia (i.e. ankle-brachial index <0.5 or toe pressure <30 mmHg)

### 2.4 Intervention: Percutaneous needle flexor tenotomy

**Fig. 1:**
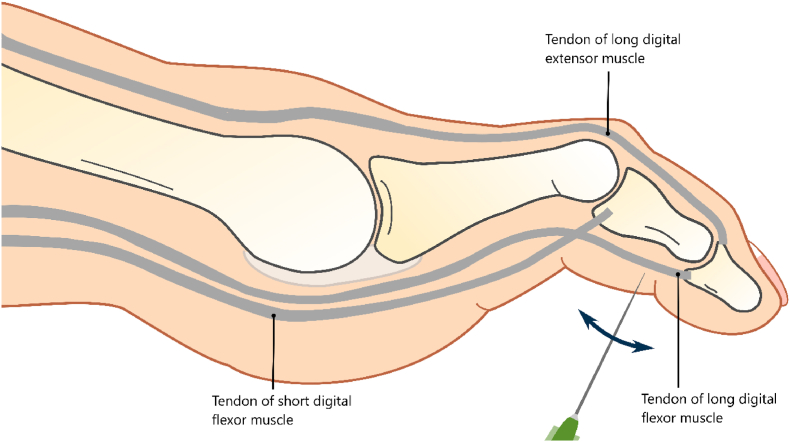
Percutaneous needle flexor tenotomy. The tendon of the long flexor muscle is severed.

Percutaneous flexor tenotomy is a minimally invasive procedure used to treat claw/hammer toe deformity [15,18,22] The foot is sanitized using chlorhexidine/alcohol and proper measures to ensure sterility are taken. In most cases, local anesthesia is not necessary due to the sensory loss in the feet of this patient group. The protective sensibility of a patient is tested before the procedure. If anesthesia is needed, local infiltration with lidocaine is used. The ankle and toe are placed in dorsiflexion to put the long digital flexor tendon under pressure. A needle is inserted at the level of the middle phalanx, making a puncture wound (Fig. 1). The tendon can be felt with the tip of the needle. Using micro movements the tendon is carefully severed (duration 1–2 min) and the needle is removed. Pressure is applied until there is no more bleeding. A bandage is placed on the wound and the subjects are advised to minimalize loading of the operated foot for 24 h. A week after the procedure the wound is checked by the treating physician. During this check, adverse events such as infection, hematomas and pain at the puncture location will be recorded.

Both the intervention group and the control group receive standard care. This includes proper wound sanitation, removing of excess callus and debridement of the ulcers by a podiatrist, evaluation of barefoot pressure measurements and in-shoe measurements of the current footwear. If necessary based on high in-shoe pressure, current footwear is adapted or new footwear is fitted by an orthopedic shoemaker and orthoses or felt are used for further off-loading.

### 2.5 Outcomes

All data and outcomes will be registered in a Castor EDC database [25]. Using this database assessors will be blinded for the outcomes.

#### 2.5.1 Ulcer recurrence

The main study outcome is ulcer recurrence on the treated toe within 2 years of follow-up. Transfer ulcers on the adjacent toes, or metatarsal heads within 2 years of follow-up will also be recorded. The formation of a claw or hammer toe in the adjacent toes will be recorded as a complication. Ulcers are defined according to the IWGDF-guideline [26]. Participants are regularly checked for ulceration by the treating physicians of the out-patient clinic or by their podiatrist.

#### 2.5.2 Toe joint angles

DIPJ, PIPJ and MTPJ angles will be measured before, and 6 and 12 months after flexor tenotomy during weight-bearing and non-weight conditions using weight-bearing CT and non-weight-bearing CT. Weight-bearing and non-weight-bearing images of both forefeet will be acquired on the Planmed Verity® CT system. This system uses cone-beam CT technology to provide 3D-images of the extremities. Subjects stand on one leg in the small bore of the weight-bearing CT scanner, with a field-of-view of approximately 13 × 16 cm. All images will be acquired subsequent to out-patient scheduled visits, therefore no additional hospital visits will be needed. In-house developed software is utilized to segment bones using region growing and manual editing where necessary [27,28]. The center of the articular surface will be computed on either side of the relevant bones. The line between these centers is used to measure the joint angles. The segmentations can be used on multiple scans of the same foot in the same patient even before and after the flexor tenotomy, using a registration technique.

#### 2.5.3 Barefoot pressure

Dynamic barefoot pressure measurements are performed using an EMED-X pressure platform (Novel GmbH, Munich, Germany). A two-step protocol with four trials per foot and a self-selected walking speed will be used. This is a reliable method to acquire pressure data without unnecessary barefoot steps [29]. The pressure distribution at the sole of the foot will be divided into 9 regions: hallux, second toe, third toe, fourth/fifth toe, metatarsal head 1, metatarsal head 2, lateral metatarsal heads, midfoot, heel. Mean peak pressure over the four steps will be calculated for each region as outcome.

#### 2.5.4 Quality of life

Quality of life is measured using the EQ-5D-5L and SF-36 questionnaires at baseline, 6 months, 12 months and 24 months. The EQ-5D-5L is a validated and extensively used tool to measure Quality of Life [30]. This questionnaire is divided in 5 dimensions: mobility, self-care, usual activities, pain/discomfort and anxiety/depression. Additionally, general quality of life is visually assed with a visual analog scale. The SF-36 is a set of questions relying on patient self-reporting [[31], [32], [33]].This tool comprises of questions relating to physical functioning, social functioning, mental health, energy, pain and perception of health.

#### 2.5.5 Cost-effectiveness

With the economic evaluation the total costs related to diabetic foot disease for all participants will be determined. These costs will be related to the effects of the treatments in the groups. The outcomes of the EQ-5D-5L and SF-36 will be used in the economic evaluation to determine cost-utility and the ulcer recurrence is used to determine cost-effectiveness. All relevant costs related to treatment will be recorded. These include.

• Cost of the flexor tenotomy
• Other costs related to prevention of recurrent ulcers prescribed at the diabetic foot rehabilitation out-patient clinic. These include: orthopedic footwear and adaptations to the footwear, felted foam, casts and orthoses
• Costs of treatment of recurrent ulcers or newly formed ulcers: wound dressing, antibiotics and treatment by podiatrist at the diabetic feet rehabilitation out-patient clinic, costs of hospitalization, interventions related to ulceration (amputation) and homecare hired due to foot ulceration
• Costs related to additional visits to the podiatrist, general practitioner, emergency department or the diabetic feet rehabilitation out-patient clinic due to diabetic foot ulcers

The costs will be valued using the guidelines published in the updated handbook for economic evaluation in the Netherlands [34]. The costs of medication will be estimated on the basis of prices charged by the Royal Society of Pharmacy.

### 2.6 Sample size calculation

For our primary outcome of ulcer recurrence, we estimate 8% recurrence in 12 months in the intervention group. This is a slightly conservative estimate, given the 11% ulcer recurrence in 53 subjects during 93 weeks of follow-up following needle tenotomy as reported by Hedegaard Andersen and colleagues [20]. In the control group, we estimate 36% ulcer recurrence in 12 months. This is slightly more positive compared to the 40% ulcer recurrence in 12 months seen in a recent review [10], and similar to the ulcer recurrence in the control group of a recently completed RCT by these authors [35]. With 8% ulcer recurrence in the intervention group and 36% in the control group, power 0.8, alpha 0.05, 1:1 randomization and intention-to-treat analysis, a total of 66 participants (33 per group) are required.

For our predefined secondary outcome of barefoot peak pressure, we estimate average barefoot peak pressure at the target toe of 400 kPa (standard deviation (SD): 250) in the control group and 180 kPa (SD: 100) in the intervention group following tenotomy. These estimates are based on clinical pilot data from our gait lab, with slightly more conservative estimates than found in our pilot study. With power 0.8, alpha 0.05, 1:1 randomization and intention-to-treat analysis, a total 40 participants (20 per group) are required for this predefined secondary outcome. With this number smaller than required for our primary outcome, the RCT can also be considered adequately powered for this outcome. The calculations were performed using clincalc.com [36].

### 2.7 Statistical analysis

Statistical analysis will be performed using SPSS. The Shapiro-Wilk W test will be used to determine the distribution. Continuous variables will be expressed as mean ± SD for normally distributed data and as median and interquartile range for not normally distributed and ordinal data. Shapiro-Wilk test and visual inspection will be performed to check for normality. In case of small sample sizes and when data is not normally distributed, Wilcoxon signed rank test will be used. Differences between groups will be compared using Fisher's Exact test or Kruskal-Wallis. A level of p < 0.05 is considered statistically significant.

## 3 Conclusion

This protocol describes a randomized controlled trial exploring the efficacy of percutaneous needle flexor tenotomy to prevent recurrent ulceration. The study will assess clinical outcomes as well as biomechanical and anatomical changes of the toes and cost-effectiveness. This will provide a comprehensive analysis of the effects of this operation. The results of the DIAFLEX trial is expected support the implementation of needle flexor tenotomy in diabetic foot care.

## Funding

M.A.M. is supported by a personal AMC-PhD scholarship 2019. M.N. is supported by a personal ZonMw VICI grant 2020 [09150182010020].

## Declaration of competing interest

The authors declare that they have no known competing financial interests or personal relationships that could have appeared to influence the work reported in this paper.

## Data Availability

No data was used for the research described in the article.
